# Immune-inflammatory biomarkers for the occurrence of MACE in patients with myocardial infarction with non-obstructive coronary arteries

**DOI:** 10.3389/fcvm.2024.1367919

**Published:** 2024-05-01

**Authors:** Hongya Zhou, Xicong Li, Wenyuan Wang, Yuanyi Zha, Guanli Gao, Silin Li, Bei Liu, Ruiwei Guo

**Affiliations:** ^1^ Department of Cardiology, Kunming Medical University, The 920th Hospital, Kunming, Yunnan, China; ^2^ Department of Cardiology, 920th Hospital of Joint Logistics Support Force, People's Liberation Army of China (PLA), Kunming, Yunnan, China; ^3^ Department of Gastroenterology, Kunming Medical University, The 920th Hospital, Kunming, Yunnan, China

**Keywords:** myocardial infarction with non-obstructive coronary arteries, inflammation, coronary artery disease, biomarker, immune inflammation

## Abstract

**Background:**

Neutrophil-to-high-density lipoprotein cholesterol ratio (NHR), monocyte-to-high-density lipoprotein cholesterol ratio (MHR), lymphocyte-to-high-density lipoprotein cholesterol ratio (LHR), platelet-to-high-density lipoprotein cholesterol ratio (PHR), systemic immune-inflammation index (SII), systemic inflammation response index (SIRI), and aggregate index of systemic inflammation (AISI) have been identified as immune-inflammatory biomarkers associated with the prognosis of cardiovascular diseases. However, the relationship of these biomarkers with the prognosis of myocardial infarction with non-obstructive coronary arteries (MINOCA) remains unclear.

**Method:**

Patients with MINOCA who underwent coronary angiography at the 920th Hospital of Joint Logistics Support Force were included in our study. Clinical baseline characteristics and laboratory testing data were collected from the hospital record system. The patients were divided into two groups on the basis of major adverse cardiovascular events (MACE) occurrence. Multiple logistic regression analysis was conducted to assess the relationship between NHR, MHR, LHR, PHR, SII, SIRI, AISI, and MACE. Receiver operating characteristic (ROC) curves were generated to evaluate the predictive value of NHR, MHR, LHR, PHR, SII, SIRI, and AISI for MACE in patients with MINOCA. The accuracy of the prediction was indicated by the area under the curve (AUC) value.

**Results:**

The study included 335 patients with MINOCA. (81 in the MACE group and 254 in the No-MACE group). The MACE group had higher levels of NHR, MHR, LHR, PHR, SII, SIRI, and AISI than the No-MACE group. Multiple logistic regression analysis adjusted for confounding factors indicated that the higher levels of NHR, MHR, PHR, SII, SIRI, and AISI were associated with the occurrence of MACE in patients with MINOCA (*P* < 0.001). The AUC values for NHR, MHR, PHR, SII, SIRI, and AISI were 0.695, 0.747, 0.674, 0.673, 0.688, and 0.676, respectively. The combination of NHR, MHR, PHR, SII, SIRI, and AISI improved the accuracy of predicting MACE in patients with MINOCA (AUC = 0.804).

**Conclusion:**

Higher levels of NHR, MHR, PHR, SII, SIRI, and AISI were associated with the occurrence of MACE, and the combination of NHR, MHR, PHR, SII, SIRI, and AISI improved the accuracy for predicting the incidence of MACE events in patients with MINOCA.

## Introduction

Cardiovascular disease (CVD) is a leading cause of death worldwide, and a major obstacle to sustainable human health (1, 2). Coronary heart disease (CHD) is the most prevalent CVD globally and causes a severe threat to human health (3, 4). Coronary angiography is considered the gold standard for diagnosing CHD and for assessing anatomical lesions in the coronary artery (5). In patients with acute myocardial infarction (AMI) undergoing coronary angiography, obstructive myocardial infarction (MI-CAD) is a common form of AMI (6, 7). However, approximately 5%–10% patients with AMI do not show significant coronary artery obstruction (normal or less than 50% narrowing of coronary arteries); this condition is termed myocardial infarction with non-obstructive coronary arteries (MINOCA) (6, 7).

MINOCA is a heterogeneous disease caused by various factors, including coronary plaque rupture, coronary artery spasm, spontaneous coronary artery dissection, and coronary artery embolism or thrombosis (7, 8). Among these factors, atherosclerotic plaque rupture is the most common one (6, 7). Although immune inflammation is not the direct cause of MINOCA, previous studies have indicated that inflammation is a crucial factor in the development of atherosclerosis, and inflammation causes endothelial injury and plaque rupture, resulting in thrombus formation (9–11). Immune inflammation is also closely associated with the prognosis of AMI (12–14). Therefore, early intervention of the immune inflammatory response may effectively reduce the incidence of adverse events in patients with MINOCA.

As immune-inflammatory biomarkers, neutrophil-to-high-density lipoprotein cholesterol ratio (NHR), monocyte-to-high-density lipoprotein cholesterol ratio (MHR), lymphocyte-to-high-density lipoprotein cholesterol ratio (LHR), platelet-to-high-density lipoprotein cholesterol ratio (PHR), systemic immune-inflammation index (SII), systemic inflammation response index (SIRI), and aggregate index of systemic inflammation (AISI) have been widely used in the prognostic assessment of diseases such as cancer and CVDs (15, 16). Currently, there is limited research on the effect of immune-inflammatory biomarkers on the prognosis of MINOCA; Patients with MINOCA had better prognosis in the risk of major long-term adverse cardiovascular events (MACE) than those with MI-CAD (17, 18). However, recent studies have shown that patients with MINOCA exhibit the same risk of MACE as those with MI-CAD (19–21). The study of Armillotta et al. showed that the rate of hospitalization for heart failure at long-term follow-up was similar between patients with MI-CAD and those with MINOCA (22). This finding suggested that patients with MINOCA deserve attention in clinical practice. The recognition of simple and practical prognostic indicators to identify high-risk patients and implement more proactive interventions not only enhances the focus on patients with MINOCA but also contributes to refining preventive strategies for MINOCA.

The main objectives of the present study were to (1) investigate the relationship between NHR, MHR, LHR, PHR, SII, SIRI, and AISI and MACE in patients with MINOCA and (2) to determine the predictive abilities of NHR, MHR, LHR, PHR, SII, SIRI, and AISI for adverse events in patients with MINOCA. The concept of our study is of noteworthy interest, since the negative prognostic role of inflammation in MINOCA patients has been observed in previous study (23).

## Materials and methods

### Study population

This study conducted a retrospective analysis of data of patients with MINOCA from the hospital record system of the 920th Hospital of Joint Logistics Support Force, People's Liberation Army of China (PLA) from January 1, 2019 to March 31, 2023 (24). Clinical baseline characteristics and laboratory testing data at baseline were collected from the hospital record system. Because the study outcome was the occurrence of MACE, information regarding MACE was collected by telephone from August 2023. The inclusion standards were as follows: (1) patients who underwent coronary angiography and (2) patients who met the diagnostic criteria for MINOCA. The exclusion criteria were as follows: (1) patients with a history of coronary revascularization treatment, including thrombolysis, percutaneous coronary intervention (PCI), or coronary artery bypass grafting; (2) patients whose coronary angiography suggested stenosis ≥50%; (3) patients with elevated levels of cardiac enzymes due to nonischemic causes such as acute myocarditis, acute heart failure, or pulmonary embolism; and (4) patients with incomplete clinical data or who were lost to follow-up.

The study followed the principles of the Declaration of Helsinki and received approval from the Ethics Committee of the 920th Hospital of Joint Logistics Support Force, PLA (approval number: 2015067). Written informed consent was obtained from the study patients.

### Diagnostic criteria for MINOCA

The diagnostic criteria for MINOCA were based on the “Fourth Universal Definition of Myocardial Infarction” (6) as follows: (1) a definitive diagnosis of AMI, defined as troponin level above the 99th percentile of the upper reference limit, and with conclusive clinical evidence of myocardial ischemia; (2) coronary angiography confirming the absence of significant coronary stenosis or stenosis <50%; and (3) exclusion of other nonischemic conditions that could cause an elevation in the level of cardiac enzymes (25).

### MACE

MACE was defined as including all-cause death, nonfatal myocardial infarction, nonfatal stroke, revascularization, and rehospitalization due to unstable angina or heart failure (26).

### Data collection

The following patient data were extracted from the hospital's electronic medical record system for analysis: patient age, gender, medical history, admission heart rate, admission blood pressure, in-hospital medication, discharge diagnosis, and laboratory results. Medical history included hypertension, diabetes, hyperlipidemia, smoking history, and stroke. In-hospital medications included dual antiplatelet therapy, beta-blockers, statins, and angiotensin-converting enzyme inhibitors/angiotensin receptor blockers. Laboratory test indicators included the levels of troponin, total cholesterol, high-density lipoprotein (HDL) cholesterol, low-density lipoprotein (LDL) cholesterol, lymphocytes, platelets, and monocytes. NHR, MHR, LHR, PHR, SII, SIRI, and AISI were calculated using the following formulas:

NHR = ratio of neutrophil count to HDL level;
MHR = ratio of monocyte count to HDL level;
LHR = ratio of lymphocyte count to HDL level;
PHR = ratio of platelet count to HDL level;
SII = platelet count multiplied by neutrophil-to-lymphocyte ratio;
SIRI = monocyte count multiplied by neutrophil-to-lymphocyte ratio;
AISI = neutrophil count multiplied by platelet count multiplied by monocyte-to-lymphocyte ratio.

### Statistical analysis

Statistical analysis was conducted using IBM SPSS Version 26.0 (IBM Corp., Armonk, NY, USA). GraphPad Prism 9 was used for graphical representation of the results. Continuous variables (metric data) that followed a normal distribution were expressed as mean ± standard deviation. The data with non-normal distribution were expressed as quartiles and interquartile range (P25–P75). Student's *t*-test or Mann–Whitney *U*-test was used to analyze baseline characteristics and laboratory examination data. Logistic regression analysis was used to investigate the relationship between clinical data, laboratory examination results, and the occurrence of MACE. Independent predictors of MACE in MINOCA patients were determined by including variables with *P* < 0.01 from univariate analysis into multivariate regression models. Receiver operating characteristic (ROC) analysis was used to predict the effect of NHR, MHR, LHR, PHR, SII, SIRI, and AISI on the occurrence of MACE in patients with MINOCA. The accuracy of the prediction was indicated by the area under the curve (AUC) value. A *P*-value of <0.05 was considered statistically significant.

## Results

### Clinical characteristics

**Figure 1:**
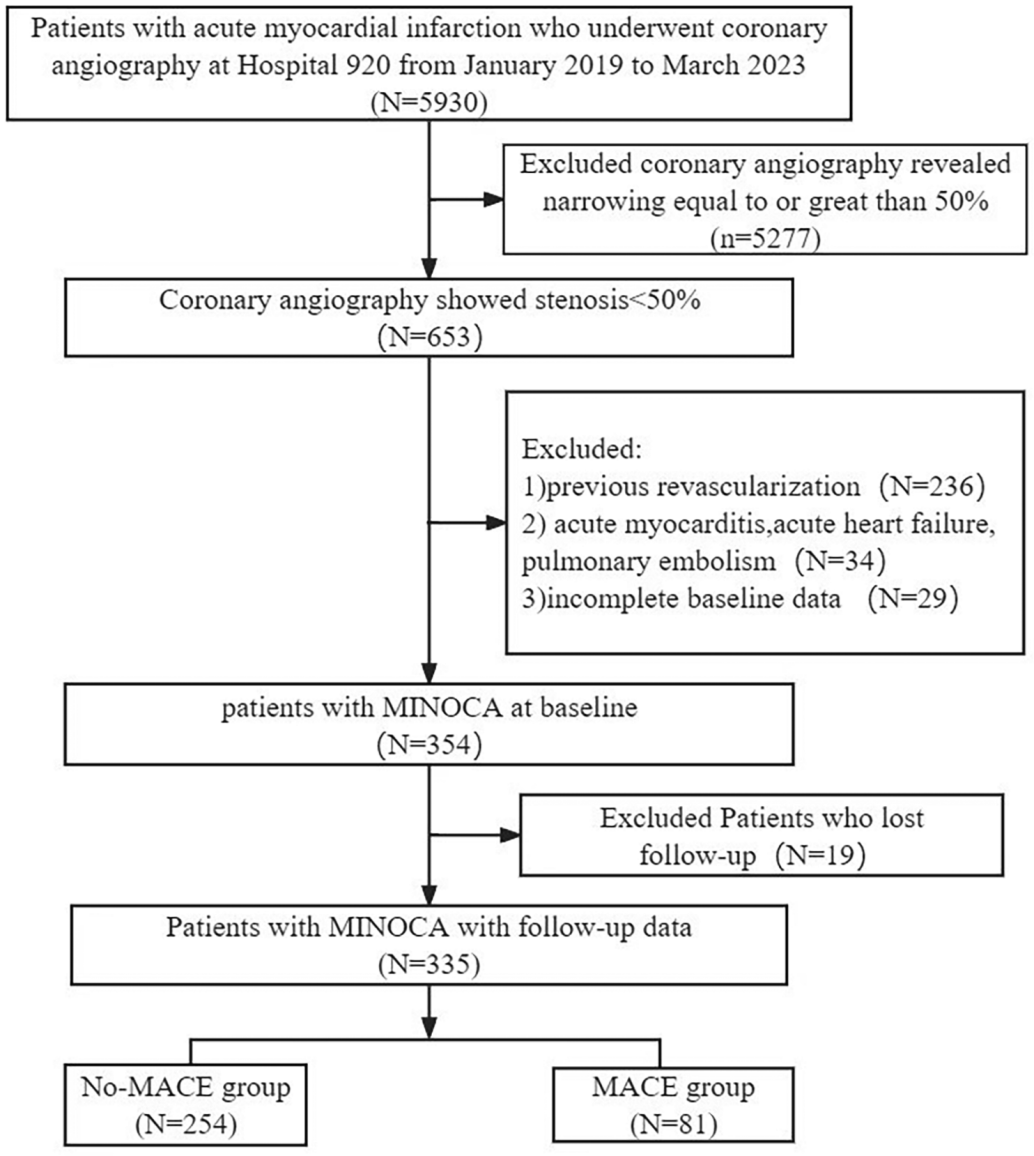
Study flowchart. Flowchart of inclusion and exclusion of the study participants. MACE, major adverse cardiovascular events; MINOCA, myocardial infarction with non-obstructive coronary arteries.

As showed in Figure 1, we selected 335 patients with MINOCA from 5,930 AMI patients who underwent coronary angiography at the 920th Hospital from January 1, 2019, to March 31, 2023. The median follow-up duration was 28 (18, 40) months. Among the 335 patients, 81 patients (24.2%) experienced MACE, and 254 patients (75.8%) did not experience MACE.

Table 1 has shown the baseline characteristics of the MACE and No-MACE groups. The average age of all study patients was 61.24 ± 12.18 years, and 56.1% patients were males. Compared to the No-MACE group, the MACE group patients were older (60.05 ± 11.81 years vs. 64.96 ± 12.63 years, *P* < 0.001), had a higher proportion of males (52.8% vs. 66.7%, *P* = 0.028), and had a higher proportion of stroke (4.3% vs. 12.3%, *P* = 0.010). No significant differences were observed between the No-MACE group and the MACE group in the proportion of hypertension, diabetes, hyperlipidemia, smoking, and in-hospital use of secondary prevention medications for CHD (all *P* > 0.05).

**Table 1:** Baseline features.

Variable	Total (*N* = 335)	No-MACE (*N* = 254)	MACE (*N* = 81)	*P* value
Age, year	61.24 ± 12.18	60.05 ± 11.81	64.96 ± 12.63	**0** **.** **001**
Male, *n* (%)	188 (56.1)	134 (52.8)	54 (66.7)	**0**.**028**
Medical history, *n* (%)				
Hypertension	109 (32.5)	81 (31.9)	28 (34.6)	0.654
Diabetes	37 (11.0)	25 (9.8)	12 (14.8)	0.214
Hypercholesterolemia	226 (67.5)	169 (66.5)	57 (70.4)	0.521
Smoking	92 (27.5)	68 (26.8)	24 (29.6)	0.616
Stroke	21 (6.3)	11 (4.3)	10 (12.3)	**0**.**010**
In-hospital medication, *n* (%)				
DAPT	165 (49.3)	119 (46.9)	46 (56.8)	0.119
Statin	200 (59.7)	150 (59.1)	49 (60.5)	0.818
ACEI or ARB	86 (25.7)	71 (28.0)	15 (18.5)	0.091
Beta-blocker	195 (58.2)	146(57.5)	49(60.5)	0.632

DAPT, dual antiplatelet therapy; ACEI, angiotensin-converting enzyme inhibitor; ARB, angiotensin receptor antagonist.

Bold values indicate significant *P* values.

### Laboratory examination

Table 2 showed differences in the laboratory examination data for all MINOCA patients and the MACE and No-MACE groups. Compared to the No-MACE group, the MACE group had lower levels of HDL cholesterol, LDL cholesterol, and lymphocytes (*P* < 0.05). Additionally, the MACE group showed significantly higher levels of creatinine, monocytes, NHR, MHR, PHR, SII, SIRI, and AISI (*P* < 0.05); however, no significant differences were observed in total cholesterol, neutrophil count, platelets, and LHR between the No-MACE and MACE groups (*P* > 0.05).

**Table 2:** Laboratory examination data.

Laboratory data	Total (*N* = 335)	No-MACE (*N* = 254)	MACE (*N* = 81)	*P* value
Creatinine	74.00 (62.00–89.00)	72.00 (61.00–88.25)	77.00 (69.00–93.50)	**0**.**009**
Total cholesterol	4.52 ± 1.19	4.56 ± 1.12	4.39 ± 1.40	0.255
HDL-C, mmol/L	1.09 (0.91–1.41)	1.18 (0.97–1.46)	0.92 (0.69–1.07)	**<0**.**001**
LDL-C, mmol/L	2.64 ± 0.92	2.70 ± 0.87	2.47 ± 1.05	**0.046**
Neutrophil, 10^9^/L	5.03 (4.07–6.00)	5.03 (4.08–5.92)	5.02 (4.05–6.57)	0.470
Lymphocyte, 10^9^/L	1.84 ± 0.56	1.95 ± 0.54	1.50 ± 0.49	**<0**.**001**
Monocyte, 10^9^/L	0.74 ± 0.23	0.72 ± 0.22	0.81 ± 0.23	**0.003**
Platelet, 10^9^/L	213.46 ± 54.17	213.02 ± 50.87	214.83 ± 63.75	0.795
NHR, 10^9^/mmol	4.41 (3.32–6.25)	4.08 (3.25–5.65)	6.01 (4.08–9.06)	**<0**.**001**
MHR, 10^9^/mmol	0.65 (0.48–0.86)	0.61 (0.45–0.77)	0.87 (0.64–1.26)	**<0**.**001**
LHR, 10^9^/mmol	1.62 (1.19–2.18)	1.62 (1.20–2.08)	1.66 (1.16–2.46)	0.551
PHR, 10^9^/mmol	192.41 (135.86–248.28)	178.95 (130.67–233.87)	232.32 (165.70–323.57)	**<0**.**001**
SII, 10^9^/L	578.72 (425.39–832.84)	552.13 (404.65–747.51)	750.35 (528.00–1,054.21)	**<0**.**001**
SIRI, 10^9^/L	2.00 (1.33–3.01)	1.86 (1.19–2.75)	2.66 (1.83–4.03)	**<0**.**001**
AISI, 10^9^/L	406.47 (274.14–630.45)	374.83 (241.67–585.40)	547.14(384.58–857.49)	**<0**.**001**

HDL-C, high-density lipoprotein cholesterol; LDL-C, low-density lipoprotein cholesterol; NHR, MHR, LHR, PHR, SII, SIRI, and AISI, were calculated using the following formulas: NHR, neutrophil count/HDL level; MHR, monocyte count/HDL level; LHR, lymphocyte count/HDL level; PHR, platelet count/HDL level; SII, platelet count × neutrophil-to-lymphocyte ratio; SIRI, monocyte count × neutrophil-to-lymphocyte ratio; AISI, neutrophil count × platelet count × monocyte-to-lymphocyte ratio.

*P* < 0.05 (two-sided) was considered statistically significant.

Bold values indicate significant *p* values.

**Figure 2:**
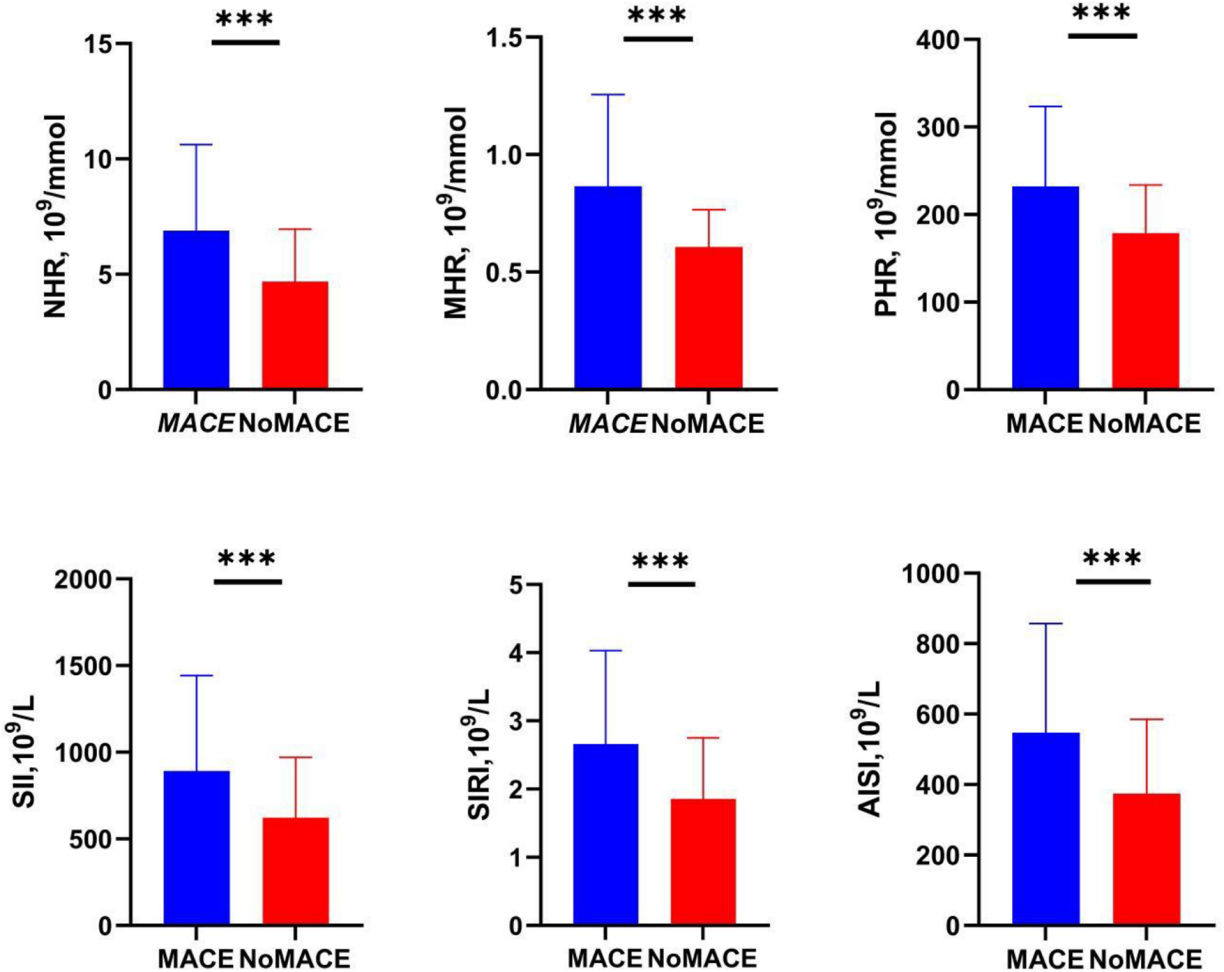
Levels of inflammatory markers in the MACE and No-MACE groups. MACE, major adverse cardiovascular events; NHR, neutrophil-to-high-density lipoprotein cholesterol ratio; MHR, monocyte-to-high-density lipoprotein cholesterol ratio; LHR, lymphocyte-to-high-density lipoprotein cholesterol ratio; PHR, platelet-to-high-density lipoprotein cholesterol ratio; SII, systemic immune-inflammation index; SIRI, systemic inflammation response index; AISI, systemic inflammation composite index. ***P < 0.0001.

As shown in the boxplot in Figure 2, compared to the No-MACE group, the MACE group had higher levels of NHR, MHR, PHR, SII, SIRI, and AISI.

### Inflammatory factors and prognosis

Table 3 showed the results of the association between laboratory examination results and the occurrence of MACE. Univariate logistic regression analysis indicated that gender, age, NHR, MHR, PHR, SII, SIRI, and AISI were associated with the occurrence of MACE. After adjusting for DAPT, statin, ACEI or ARB, beta-blocker, creatinine levels, total cholesterol and LHR, variables with statistical significance (*P* < 0.05) were included in the multivariate logistic regression analysis model. Gender, age, NHR, PHR, SIRI, and AISI were still significantly associated with the occurrence of MACE (*P* < 0.05).

**Table 3:** Correlation between inflammatory factors and MACE in patients with MINOCA.

Variable	Unadjusted	*P* value	Adjusted	*P* value
	Variable OR (95% CI)		Variable OR (95% CI)	
Age	1.036 (1.013–1.059)	<**0**.**001**	1.040 (1.013–1.067)	<**0.001**
Male	0.558 (0.331–0.942)	**0.030**	0.421 (0.218–0.812)	**0.010**
DAPT	1.491 (0.901–2.468)	0.120	—	—
Statin	1.062 (0.637–1.770)	0.820	—	—
ACEI or ARB	0.586 (0.314–1.093)	0.090	—	—
Beta-blocker	1.133 (0.680–1.887)	0.630	—	—
Creatinine	1.001 (0.998–1.003)	0.726	—	—
Total cholesterol	0.882 (0.711–1.095)	0.250	—	—
NHR, 10^9^/mmol	1.299 (1.183–1.426)	<**0**.**001**	0.807 (0.671–0.971)	0.020
MHR, 10^9^/mmol	18.577 (7.700–44.821)	<**0**.**001**	5.248 (0.547–50.312)	0.150
LHR, 10^9^/mmol	1.224 (0.874–1.713)	0.240	—	—
PHR, 10^9^/mmol	1.008 (1.005–1.011)	<**0**.**001**	1.012 (1.004–1.020)	<**0.001**
SII, 10^9^/L	1.001 (1.001–1.002)	<**0**.**001**	1.002 (0.999–1.005)	0.240
SIRI, 10^9^/L	1.415 (1.225–1.635)	<**0**.**001**	2.535 (1.372–4.686)	<**0.001**
AISI, 10^9^/L	1.001 (1.001–1.002)	<**0**.**001**	0.996 (0.993–0.999)	<**0.001**

MACE, major adverse cardiovascular events; DAPT, dual antiplatelet therapy; ACEI, angiotensin-converting enzyme inhibitor; ARB, angiotensin receptor antagonist.

DAPT, statin, ACEI or ARB, beta-blocker, creatinine levels, total cholesterol and LHR were adjusted.

*P* < 0.05 was considered statistically significant.

Bold values indicate significant *p* values.

### Predictive value of inflammatory factors

**Figure 3:**
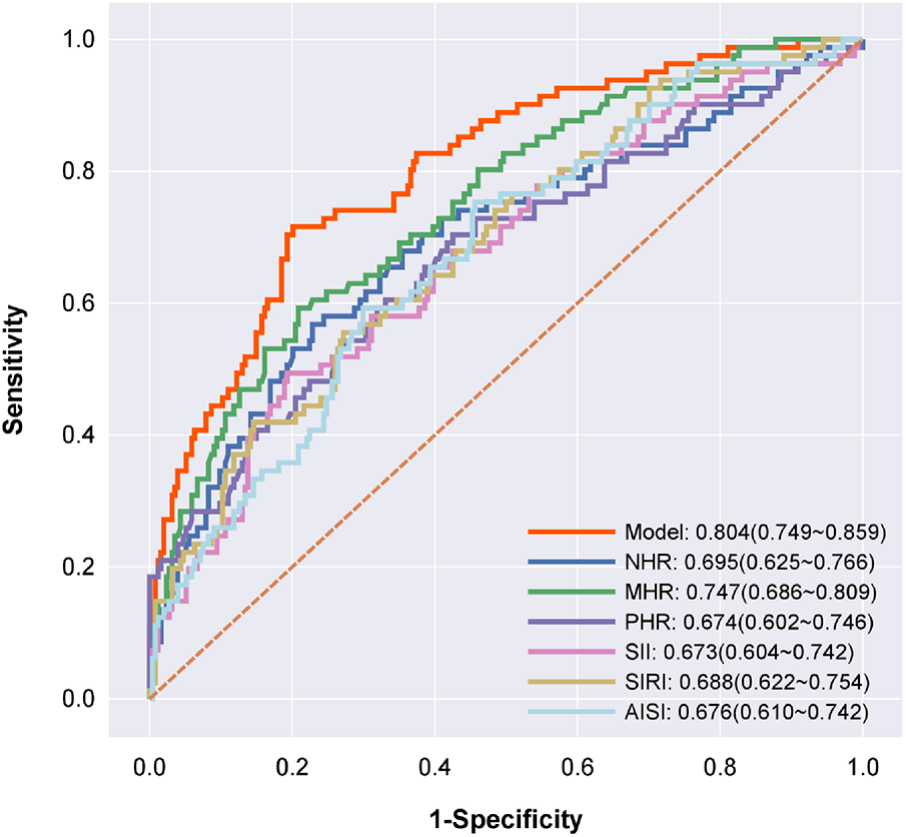
Receiver operating characteristic curve analyses for predicting major adverse cardiovascular events. Receiver operating characteristic curve analyses for predicting major adverse cardiovascular events. The model (red) includes NHR + MHR + PHR + SII + SIRI + AISI; NHR (dark blue); MHR (green); PHR (violet); SII (pink); SIRI (yellow); and AISI (baby blue), *P* < 0.001.

ROC curve analysis was used to assess the ability of NHR, MHR, PHR, SII, SIRI and AISI to predict MACE occurrence in patients with MINOCA. The results showed that these inflammatory factors had a high predictive value for MACE occurrence in MINOCA patients as follows NHR: AUC = 0.695, 95% CI = 0.625–0.766, *P* = 0.000; MHR: AUC = 0.747, 95% CI = 0.686–0.809, *P* = 0.000; PHR: AUC = 0.674, 95% CI = 0.602–0.746, *P* = 0.000; SII: AUC = 0.673, 95% CI = 0.604–0.742, *P* = 0.000; SIRI: AUC = 0.688, 95% CI = 0.622–0.754, *P* = 0.000; AISI: AUC = 0.676, 95% CI = 0.610–0.742, *P* = 0.000. The AUC value for predicting MACE in patients with MINOCA by using the combination of NHR + MHR + PHR + SII + SIRI + AISI was 0.804 (95% CI = 0.749–0.859). Therefore, compared to a single index, the combined indicator significantly improved the diagnostic efficiency of predicting the occurrence of MACE (Figure 3).

## Discussion

This study found that higher levels of NHR, MHR, PHR, SII, SIRI, and AISI were associated with the occurrence of MACE, and the combination of NHR, MHR, PHR, SII, SIRI, and AISI improved the accuracy for predicting the incidence of MACE events in patients with MINOCA.

MINOCA is a special type of AMI wherein myocardial infarction is present without significant obstruction of the coronary artery (6). Although patients with MINOCA do not have coronary artery blockages, they are still at a relatively high risk of onset of adverse cardiovascular events (19, 21). Therefore, early risk stratification for MINOCA patients is critical to identify high-risk individuals and implement more proactive and decisive intervention measures (27). We found that patients with MINOCA constituted 5.6% of patients with AMI. A previous study showed that MINOCA accounts for 1%–13% of AMI patients (7). A meta-analysis indicated that the incidence of MINOCA in patients with MI was 6% (28), which was similar to the findings of the present study. This meta-analysis also found that 40% of patients with MINOCA were females. Our study had a similar conclusion, with males comprising 56.1% of all patients with MINOCA. Thus, there were more males than females among patients with MINOCA in our study. MINOCA is categorized as a CVD, and inflammation plays a crucial role in the development and progression of CVDs. Inflammatory reactions can lead to the formation and rupture of atherosclerotic plaques, thereby triggering severe cardiovascular events such as myocardial infarction and stroke (29). Atherosclerosis was considered a cholesterol storage disease characterized by the accumulation of cholesterol and thrombus fragments in the arterial wall (30). However, recent studies have shown the proliferation of smooth muscle cells (SMCs) in atherosclerotic plaque lesions (30, 31). In recent years, the significance of inflammation in the development of atherosclerosis has gained increasing recognition. Inflammation has been proposed as a possible trigger also in patients with spontaneous coronary artery dissection, which occurs more frequently in women and can cause myocardial infarction and sudden cardiac death, and differential diagnosis with atherosclerotic MINOCA is crucial, since therapy is totally different, but the role of anti-inflammatory drugs remains uncertain (32, 33).

Atherosclerosis is currently regarded as a chronic inflammatory disease of the arterial wall (34), characterized by immune-inflammatory dysfunction involving interactions between immune cells and vascular cells (11, 35). Monocytes initiate intracellular lipid accumulation by releasing proinflammatory cytokines, reactive oxygen species, and proteolytic enzymes, thereby promoting fibrous cap fragility and the formation of lipid core thrombi (10, 36). Neutrophils exacerbate tissue damage and inflammation in the late stages of atherosclerosis by triggering the lysis and death of SMCs (37). Conversely, lymphocytes may impede the progression of atherosclerosis, thereby playing a protective role (38). Platelets have a dual role in atherosclerosis: platelet adhesion to the endothelial surface may signal the recruitment and extravasation of monocytes, thereby playing a critical role in the initiation of atherosclerotic plaque formation (39), and platelet activation can initiate thrombus formation, which promotes blood clotting (40). Elevated LDL and reduced HDL levels are the crucial factors in the occurrence and progression of atherosclerosis (41, 42). Therefore, understanding the relationship between inflammation and CVDs is very critical to prevent and treat CVDs. Recent studies have suggested that NHR (15), MHR (43, 44), LHR (45), PHR (46), SII (47), SIRI (48), and AISI (49) are novel inflammatory markers with significant clinical relevance in the occurrence, development, and prognosis of many diseases because of their ease of acquisition. NHR (15) can serve as a predictive indicator for the long-term clinical outcomes of elderly patients with AMI, exceeding MHR and LDL-C/HDL-C. Higher MHR and SIRI levels were associated with an increased risk of metabolic diseases and CVD. Low LHR (49) can serve as a mortality predictor in septic patients. PHR has a high predictive ability in diagnosing and assessing metabolic syndrome and could serve as a biomarker for diagnosing the risk of atherosclerotic thrombosis (46). SII and SIRI are significantly correlated with MACE occurrence in patients with AMI and thus could serve as useful indicators to predict MACE (16). In adults with hypertension, an elevated AISI *value* was significantly associated with an increased risk of CVD-related death, thus functioning as an early warning parameter for adverse outcomes (50).

Our study further extends the role of inflammatory biomarkers to patients with MINOCA. We found that the MACE group had higher levels of inflammatory markers than the No-MACE group, and high levels of inflammatory markers were independent risk factors for MACE in patients with MINOCA. We further assessed the diagnostic value of inflammatory markers in predicting MACE in patients with MINOCA. We found that NHR, MHR, PHR, SII, SIRI, and AISI could predict the occurrence of MACE in patients with MINOCA, with AUC values of 0.695, 0.747, 0.674, 0.673, 0.688, and 0.676, respectively. We further integrated these six inflammatory markers into a model and found that the integrated model could enhance the diagnostic value of predicting MACE in patients with MINOCA, with an AUC value of 0.804. This finding indicates that the use of the combined model in clinical practice can better predict the occurrence of MACE in patients with MINOCA, thus providing convenient diagnostic assistance for clinicians. Moreover this study confirms that the prognosis of MINOCA patients is not benign, especially in high-risk subgroups such as patients with positive inflammation markers of with unmet secondary prevention targets (51).

According to the findings of the present study, future research can further explore the relationship between NHR, MHR, PHR, SII, SIRI, and AISI with specific MACE outcomes, such as causes of death and mortality rates. Additionally, it would be worthwhile to consider integrating these inflammatory biomarkers with other predictive models or scoring systems to enhance the accuracy of prognostic assessment for patients with MINOCA.

### Limitations

There are some limitations in this study. First, this study analyzed only the overall MACE events in patients with MINOCA and did not specifically examine the factors leading to MACE events, such as reasons for rehospitalization, specific causes of death, and time of death. Second, the retrospective study design may have introduced information bias. Third, the study sample was drawn from a single center, and although it possesses a degree of representativeness, it may not be fully representative of the overall population. Additionally, cardiac magnetic resonance (CMR) is crucial to identify whether the underlying mechanism of acute myocardial injury is ischemic or non-ischemic and is related to cardiomyopathy, myocarditis, or Takotsubo syndrome) (52, 53); however, this study was based only on the clinician's experience and many patients did not undergo CMR examination for identifying the underlying mechanism of acute myocardial injury, and it is possible that some myocarditis have been included in the population, thus affecting results (54). Left ventricular ejection fraction (LVEF) and renal failure are critical indicators of cardiovascular outcomes. C-reactive protein (CRP) is also a common biomarker of inflammation. Because of the small database, the data regarding LVEF, renal failure, and CRP were unavailable; consequently, these indicators were not included in the present study and require further research in future investigations. Finally, the sample size of this study was relatively small, and it was designed as a cross-sectional study. Thus, a cause-and-effect relationship cannot be established, and further long-term follow-up studies are required to confirm the stability and reliability of the conclusions.

## Conclusion

The present study revealed correlation between NHR, MHR, PHR, SII, SIRI, and AISI with MACE occurrence in patients with MINOCA. Higher levels of inflammatory biomarkers were associated with a higher risk of MACE. The combination model was more valuable in predicting MACE events in patients with MINOCA. This study has reported a new predictive method for patients with MINOCA, which could enable physicians to more accurately assess disease risk in patients. In practical terms, our research has provided clinicians a simple, cost-effective, and noninvasive approach for screening and predicting the risk of MACE occurrence in patients with MINOCA. This can aid in early intervention and treatment, eventually improving the prognosis of patients with MINOCA.

## Data Availability

The raw data supporting the conclusions of this article will be made available by the authors, without undue reservation.
